# Supplementary material for: Training diversity promotes absolute-value-guided choice
Source: PLoS Comput Biol. 2022 Nov 2;18(11):e1010664. doi: 10.1371/journal.pcbi.1010664 (PMC9678339; doi:10.1371/journal.pcbi.1010664)
Supplement: S1 Table — (DOCX) [file pcbi.1010664.s001.docx]

**S1 Table. Performance on testing trials.**

**Learned-pair test**

|  | **Mean** | **SE** | **Bootstrap-test** |
| --- | --- | --- | --- |
| Low concurrent | .87 | .02 |  |
| High concurrent | .86 | .02 | p=.42 |
|  |  |  |  |
| Low cumulative | .88 | .01 |  |
| High cumulative | .85 | .01 | p=.20 |

**Novel-pair test**

|  | **Mean** | **SE** | **Bootstrap-test** |
| --- | --- | --- | --- |
| Low concurrent | .82 | .01 |  |
| High concurrent | .84 | .01 | p=.10 |
|  |  |  |  |
| Low cumulative | .85 | .01 |  |
| High cumulative | .80 | .01 | p=.01 |
