# Supplementary material for: Training diversity promotes absolute-value-guided choice
Source: PLoS Comput Biol. 2022 Nov 2;18(11):e1010664. doi: 10.1371/journal.pcbi.1010664 (PMC9678339; doi:10.1371/journal.pcbi.1010664)
Supplement: S2 Table — (DOCX) [file pcbi.1010664.s002.docx]

**S2 Table. Bayesian logistic regression of subjects’ choices on reward history, training diversity, and past choice.**

|  | Estimate | Est. Error | l-95% CI | u-95% CI |
| --- | --- | --- | --- | --- |
| $\beta_{0}$ | -0.02 | 0.04 | -0.10 | 0.06 |
| $\beta_{Own}$ | 1.97 | .16 | 1.63 | 2.31 |
| $\beta_{Current alternative}$ | -.43 | .11 | -.64 | -.21 |
| $\beta_{\mathrm{Other}}$ | -.53 | .10 | -.75 | -.32 |
| $\beta_{\mathrm{Concurrent}}$ | .01 | .02 | -.04 | .05 |
| $\beta_{Times Chosen}$ | .04 | .01 | .04 | .05 |
| $\beta_{Concurrent \times Own}$ | .17 | .09 | -.10 | .44 |
| $\beta_{Concurrent \times Current alternative}$ | .06 | .06 | -.08 | .20 |
| $\beta_{Concurrent \times Other}$ | .39 | .05 | .30 | .49 |
| $\beta_{\mathrm{Cumulative}}$ | -.01 | .02 | -.04 | .02 |
| $\beta_{Cumulative \times Own}$ | -.21 | .07 | -.34 | -.06 |
| $\beta_{Cumulative \times Current alternative}$ | -.06 | .06 | -0.17 | .05 |
| $\beta_{Cumulative \times Other}$ | .11 | .07 | -.02 | .23 |
