# Supplementary material for: Training diversity promotes absolute-value-guided choice
Source: PLoS Comput Biol. 2022 Nov 2;18(11):e1010664. doi: 10.1371/journal.pcbi.1010664 (PMC9678339; doi:10.1371/journal.pcbi.1010664)
Supplement: S3 Table — 10 full experimental datasets were simulated using each model. Rows indicate the model used to simulate data and columns indicate the model recovered from the data using the model comparison procedure. (DOCX) [file pcbi.1010664.s003.docx]

**S3 Table. Model validation.** 10 full experimental datasets were simulated using each model. Rows indicate the model used to simulate data and columns indicate the model recovered from the data using the model comparison procedure.

|  | **Preference+Value** | **Value** | **Preference** |
| --- | --- | --- | --- |
| **Preference+Value** | 10 | 0 | 0 |
| **Value** | 0 | 10 | 0 |
| **Preference** | 0 | 0 | 10 |
