# Supplementary material for: Training diversity promotes absolute-value-guided choice
Source: PLoS Comput Biol. 2022 Nov 2;18(11):e1010664. doi: 10.1371/journal.pcbi.1010664 (PMC9678339; doi:10.1371/journal.pcbi.1010664)
Supplement: S4 Table — We validated our parameter fits through simulating data using the best fitting parameters for each subject and then recovering those parameters. Our correlation between simulated and recovered parameters was at least .74 for all parametes of interest that capture the effects of the experimental conditions, and at least .51 for all other parameters. (DOCX) [file pcbi.1010664.s004.docx]

**S4 Table. Validation of Parameter Recovery.** We validated our parameter fits through simulating data using the best fitting parameters for each subject and then recovering those parameters. Our correlation between simulated and recovered parameters was at least .74 for all parameters of interest that capture the effects of the experimental conditions, and at least .51 for all other parameters.

| **Parameter** | **Correlation between Simulated and Recovered Parameters** |
| --- | --- |
| $\beta_{value baseline}$ | 0.96 |
| $\beta_{Preference baseline}$ | 0.97 |
| $\beta_{\boldsymbol{test}}$ | 0.71 |
| $\beta_{concurrent}$ | 0.54 |
| $\beta_{cumulative}$ | 0.64 |
| $\beta_{\boldsymbol{concurrent x value}}$ | 0.93 |
| $\beta_{cumulative x value}$ | 0.74 |
| $\beta_{\boldsymbol{concurrent x Test}}$ | 0.87 |
| $\beta_{Value x Test}$ | 0.73 |
| $\gamma_{Value}$ | 0.91 |
| $\gamma_{Preference}$ | 0.61 |
