# Supplementary material for: Training diversity promotes absolute-value-guided choice
Source: PLoS Comput Biol. 2022 Nov 2;18(11):e1010664. doi: 10.1371/journal.pcbi.1010664 (PMC9678339; doi:10.1371/journal.pcbi.1010664)
Supplement: S5 Table — To validate the BIC model comparison results, we also performed model comparison using the Akaike information criterion (AIC). (DOCX) [file pcbi.1010664.s005.docx]

**Table S5: Model comparison using AIC.** To validate the BIC model comparison results, we also performed model comparison using the Akaike information criterion (AIC).

|  | **AIC** | **BIC** |
| --- | --- | --- |
| **Winning model** | 56,576 | 56,854 |
| **Value-only model** | 59,352 | 59,482 |
| **Preference-only model** | 66,476 | 66,624 |
| **RW variant of the winning model** | 57,478 | 57,755 |
