# Supplementary figures and images for: Training diversity promotes absolute-value-guided choice
Source: PLoS Comput Biol. 2022 Nov 2;18(11):e1010664. doi: 10.1371/journal.pcbi.1010664 (PMC9678339; doi:10.1371/journal.pcbi.1010664)

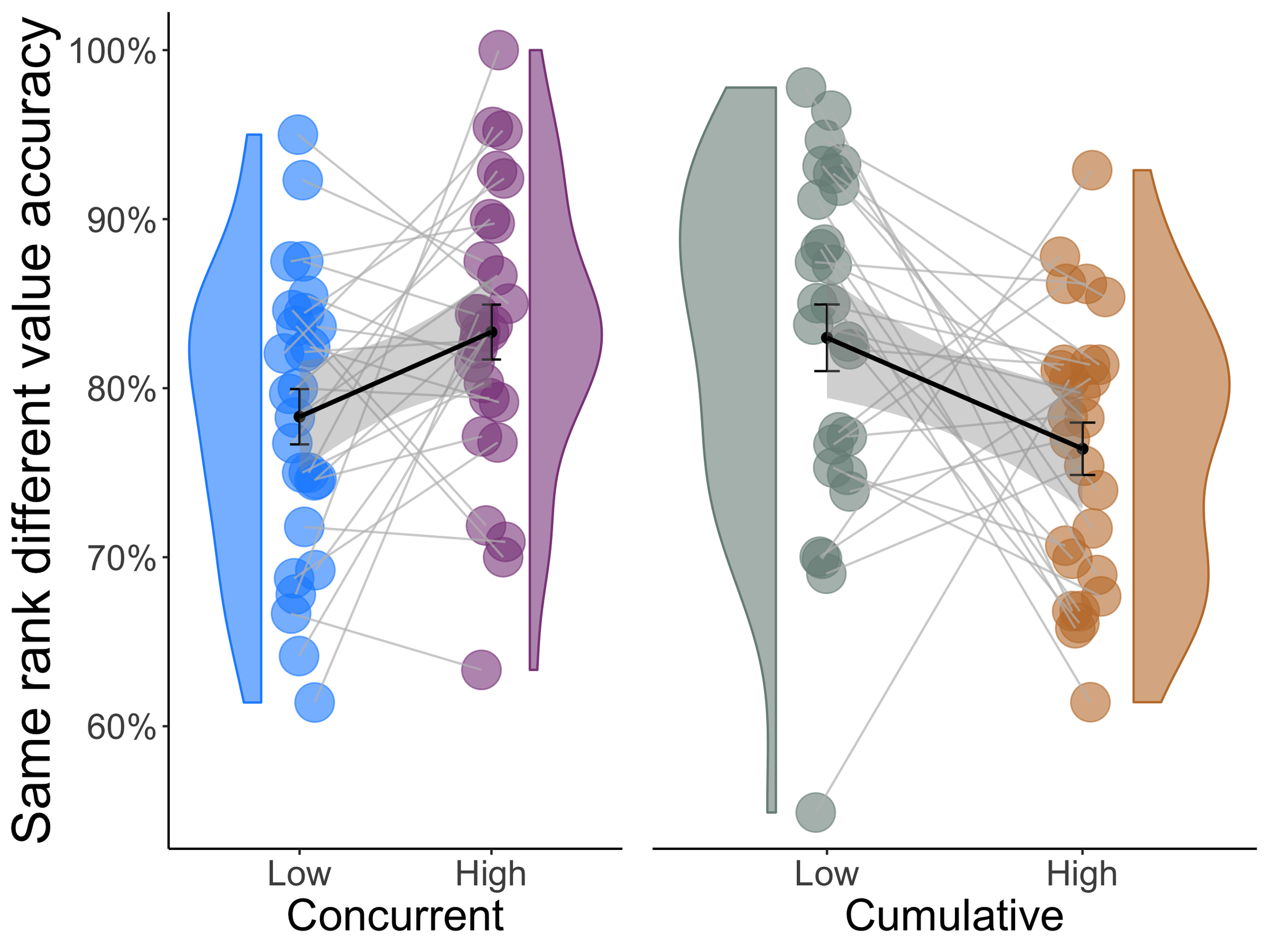

Supplement: S1 Fig — Concurrent diversity improved subjects’ performance (Mean_high = .83±.02; Mean_low = .78±.02; pcorrected = .035 bootstrap test). By contrast, cumulative diversity impaired performance on such trials (Mean_low = .82±.02 Mean_high = .77±.02 pcorrected = .04 bootstrap test), as consistent with its general effect on overall performance. The plot shows individual subject accuracy (circles), group distributions of accuracy levels (violin), group means (thick lines) and standard errors (gray shading). (TIF) [file pcbi.1010664.s006.tif]

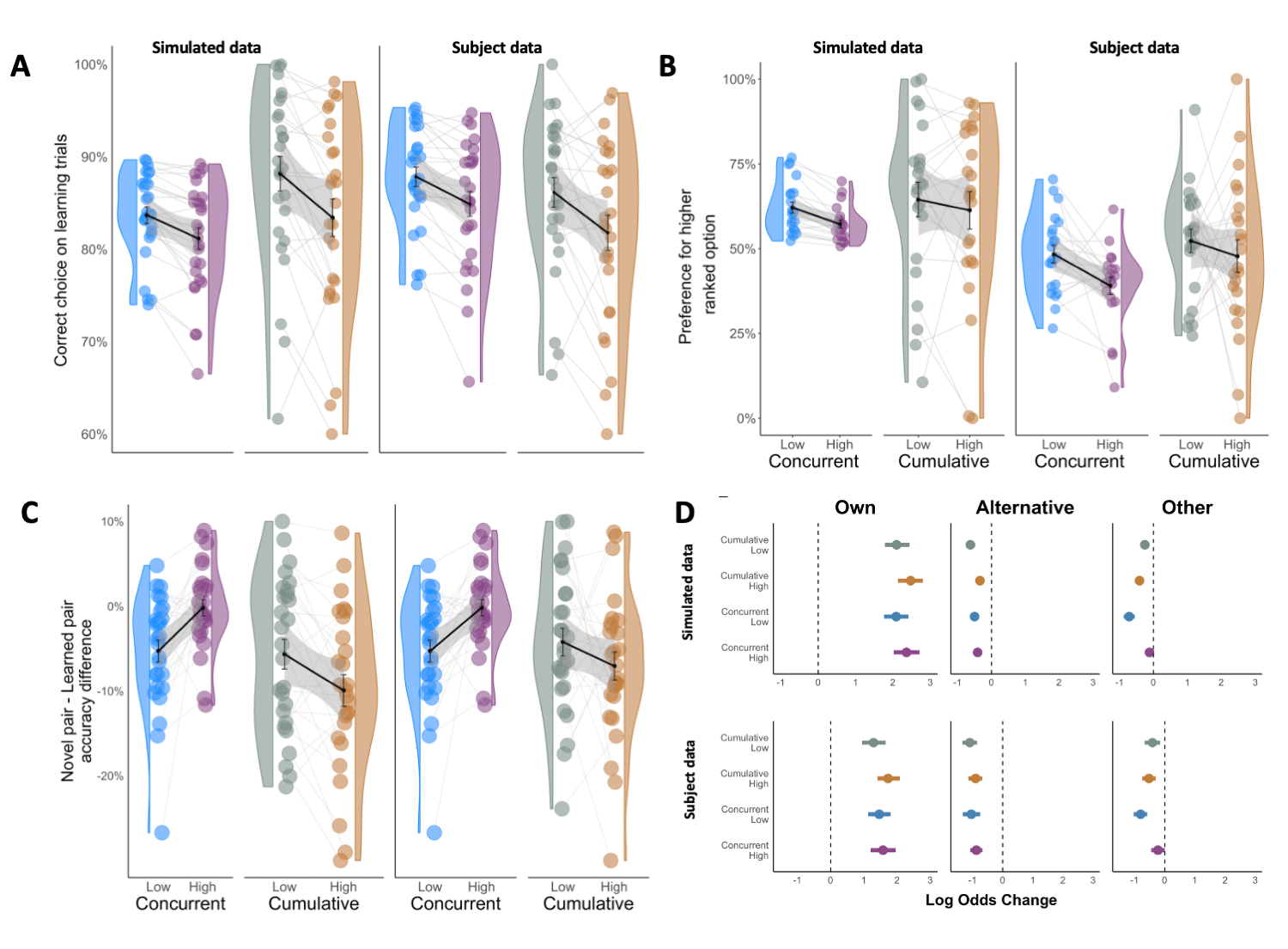

Supplement: S2 Fig — To determine whether the model successfully captured individual differences in our experiment, we examined how parameter fits correlated with model-agnostic measures of behavior. As expected, we found that βvalue was significantly correlated with generalization performance (r = .7) while βpreference correlated with our measure of rank bias (r = .5). We then validated the best-fitting model thoroughly by simulating, for each subject, 1000 data sets using their best fitting parameters and analyzing the simulated data in the same fashion in which we analyzed the real data. This procedure showed the model uniquely accounted for all of our behavioral findings (Fig 1) (A) In learning trials, performance is better in conditions of low concurrent (Meanlow = 88% ±1% vs Meanhigh = 85%±1) and cumulative (Meanlow = 85%±1% vs Meanhigh = 82%±1%) diversity. (B) Concurrent but not cumulative diversity leads to better generalization (Concurrent: Meanlow = −7.8%±1% vs Meanhigh = −1.2%±1 pcorrected < .001 bootstrap test; Cumulative Meanlow = −4%±1% vs Meanhigh = −6%±1 pcorrected = .23). (C) Concurrent but not cumulative diversity diminishes ranking bias (Concurrent: Meanlow = 59% ±1% vs Meanhigh = 53%±1, p = .01, Cumulative: Meanlow = 56% ±1% vs Meanhigh = 54%±1). (D) The simulated choices show that preference for an image is inversely influenced by the outcomes of both the current alternative (βCurrent alternative = -.46, CI = [-.56, -.35]) and of the other images it had previously been pitted against (βOther = -.42, CI = [-.54, -.30]). Furthermore, the influence of other images’ reward history is reduced by high concurrent diversity (βconcurrent×other = .31, CI = [.22, .40]). (TIF) [file pcbi.1010664.s007.tif]

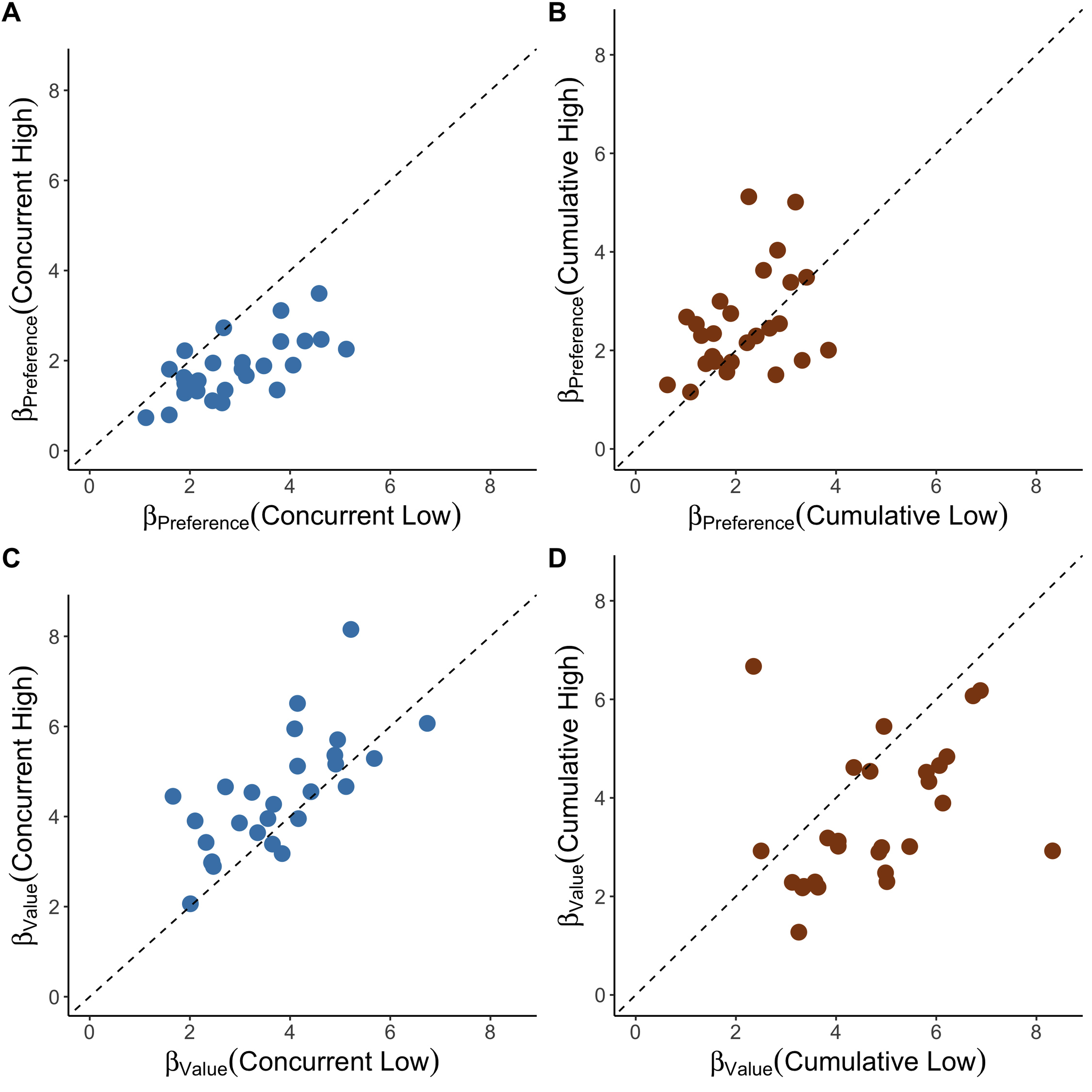

Supplement: S3 Fig — To account for previous findings of asymmetric in learning from positive versus negative reward prediction errors [54] we implemented a beta binomial model with asymmetric update rates. This modification did not alter any of the main findings. Namely, preference learning manifested to a greater extent in conditions of low concurrent diversity (low concurrent: βpreference = 3.07 ±.21; high concurrent: βpreference = 1.76 ±.12; p<.001 permutation test) whereas value learning manifested to a greater extent in conditions of high concurrent diversity (low concurrent: βvalue = 4.04±.18; high concurrent: βvalue = 4.54±.14 p<.001 permutation test). Furthermore, as in our winning model, cumulative diversity inhibited value learning (low cumulative: = 4.99 ±.3; high cumulative: βvalue = 3.78 ±.3; p < .001 permutation test) and had no significant impact on preference learning (low cumulative: βpreference = 1.60±.12; high cumulative: βpreference = 1.87±.14; p = .11 permutation test) (TIF) [file pcbi.1010664.s008.tif]
