# Supplementary material for: Training diversity promotes absolute-value-guided choice
Source: PLoS Comput Biol. 2022 Nov 2;18(11):e1010664. doi: 10.1371/journal.pcbi.1010664 (PMC9678339; doi:10.1371/journal.pcbi.1010664)
Supplement: Attachment — Submitted filename: Responses_to_reviewers.docx [file pcbi.1010664.s009.docx]

## *(note- line numbers are in reference to the clean version of the revised manuscript and not to the version with tracked changes).*

## Reviewer 1

**Comment 1.1:** Thank you for the opportunity to review this manuscript by Solomyak and colleagues, which reports the results of an interesting longitudinal experiment studying the factors that influence absolute-value-guided choice versus relative-value-guided choice. This topic speaks to a recent debate in the reinforcement learning literature, which distinguishes between (absolute-)value-based learning models, in which participants are assumed to learn a representation of the discounted cumulative expected reward associated with different options, versus relative-value (or preference-based) learning models, in which participants learn a representation of the relative goodness of different choice options, but not their absolute value. This manuscript argues that one situational factor that promotes learning of absolute values is 'concurrent diversity', operationalized as the number of different alternatives that a choice option is paired with during the learning phase.

Strengths of the paper include its careful longitudinal research design and its use of sophisticated modelling analyses to get at the aspects of the data that address its research question. I have several questions about the manuscript that are mostly about ascertaining that the statistical analyses do indeed provide an appropriate degree of support for the manuscript's conclusions.

**Response:** We thank the reviewer for their overall favorable appraisal of our work, and for their valuable suggestions which have helped bolster the study’s conclusion and improve the clarity of the methods.

**Comment 1.2:** One of the primary model-agnostic statistics in support of the manuscript's conclusions (reported on Page 14, lines 176-182) is that at the test phase, participants preferred similarly rewarding higher-ranked images from low-concurrent-diversity conditions (Mean = 63%, p = .006) but not from the high-concurrent-diversity condition (Mean = 49%, p = .24). First of all, these numbers should be tested against one another to ensure that this difference is statistically significant. Secondly, my impression is that this test does not account for the actual reward rates of the different stimuli (i.e., the higher-ranked image might have a reward rate of 65% and the lower-ranked image might have a reward rate of 56%). It is therefore crucial to control for the actual reward rate differences of the two stimuli in the test phase in this analysis, and to show that the difference between these two conditions remains statistically significant once the actual reward rate differences of the two stimuli are accounted for.

**Response:** We agree with the reviewer about the weaknesses of this analysis. Precisely for this reason, this analysis primarily serves for visualizing the data, whereas conclusions are drawn based on the regression analysis that immediately follows (lines 200-211). This latter analysis indeed controls for the actual reward rates of the different stimuli, and in addition makes use of more of the data. Thus, it offers a more valid and sensitive test of participants’ preference for higher-ranking images. Nevertheless, for completeness we now also report the results of a direct comparison between the raw numbers and report as follows (line 189-190): *This difference between conditions trended towards significance (p=.06 permutation test) and was not evident as a function of cumulative diversity (Mean low=57% ±7%, Mean high=48% ±8%).*

Furthermore, we have now edited the presentation of the results to better clarify their roles and limitations (lines 185-199, new changes in **bold**):

***Visualizing*** *choices between similarly rewarded images (i.e. less than 10% difference in reward rate) showed that subjects preferred images that ranked higher during learning (i.e., that were pitted against images with lower reward probabilities) under low concurrent diversity (Mean_low_=63% SEM* $\pm6\%,$ *with 50% representing no preference between images) but not under high concurrent diversity (Mean_high_=49% SEM* $\pm5\%$*; Figure 4A).* ***This difference between conditions trended towards significance (p=.06 permutation test) and was not evident as a function of cumulative diversity (Mean low=57% ±7%, Mean high=48% ±8%)****.*

*The above measure of rank bias, however, is limited in both sensitivity and validity. First, it ignores the random differences that inevitably exist in the actual outcomes of similarly ranked images. Second, it does not utilize the information that exists in subjects’ choices between differently ranked images. Third, it is confounded by the fact that higher ranking images were chosen more during learning, since they were pitted against less rewarding images. This latter confound is important because it means that more outcomes were observed for higher-ranking images, which may have allowed subjects to develop greater confidence regarding their value.*

We then go on to report the results of the Bayesian logistic regression analysis which directly addresses these limitations (lines 200-211).

*To address these challenges, we used a Bayesian logistic mixed model that predicted subjects’ choices based on differences between the currently available images’ own reward history (*$\beta_{Own};$ *i.e., proportion rewarded), the number of times each image was chosen (*$\beta_{Times Chosen}$ *i.e., sampling bias), and the reward history from trials in which the currently available images were rejected in favor of other images (Figure 4B). The latter was separated into two regressors, for prior outcomes of rejecting a presently available image in favor of the other presently available image (*$\beta_{Current alternative}$*) and of rejecting it in favor of any other image (*$\beta_{Other})$*. Importantly, only the latter regressor unequivocally captures the effect of other images’ outcomes that are irrelevant for maximizing absolute expected value. To determine whether the effects of different types of outcomes was modulated by training diversity, we included regressors for concurrent and cumulative diversity and interactions between both types of diversity and each type of reward history.*

**Comment 1.3:** The beta-binomial model implicitly assumes that participants learn equivalently from gains and losses (Equation 1 and Equation 2). Other beta-binomial models are possible which allow for asymmetries in the learning process (e.g., the count of reward outcomes may be incremented at a different rate to the count of non-reward outcomes). Given the numerous studies showing asymmetries in learning from positive versus negative reward prediction errors (including in a transfer learning context; see Ciranka et al., Nature Human Behaviour, 2022), it could be useful to include such an asymmetry in the learning models.

**Response:** We thank the reviewer for the suggestion to consider learning asymmetries in the beta-binomial models. We have now implemented this modification, by differentially incrementing counts as described by the reviewer, and found that it indeed improved the fit to the data. Reassuringly, this modification did not alter any of the main findings. This analysis is now reported in the main text (line 757-759) and shown in Supplementary Figure S3:

*Finally, we tested a beta-binomial model which allowed for asymmetries in the learning process from rewarded and non-rewarded outcomes. This model indeed improved the fit to the data but did not alter any of the main findings (Figure S3)*

**Figure S3. Model fits of the winning model with asymmetric learning rates.** To account for previous findings of asymmetric in learning from positive versus negative reward prediction errors (Ciranka et. al. 2022) we implemented a beta binomial model with asymmetric update rates. This modification did not alter any of the main findings. Namely, preference learning manifested to a greater extent in conditions of low concurrent diversity (low concurrent: $\beta_{\mathrm{preference}}$=3.07 $\pm.21$; high concurrent: $\beta_{\mathrm{preference}}$=1.76 $\pm.12$; $p<.001$ permutation test) whereas value learning manifested to a greater extent in conditions of high concurrent diversity (low concurrent: $\beta_{\mathrm{value}}=4.04$ $\pm.18$; high concurrent: $\beta_{\mathrm{value}}=4.54 \pm.14$ $p<.001$ permutation test). Furthermore, as in our winning model, cumulative diversity inhibited value learning (low cumulative: =4.99 $\pm.3$; high cumulative: $\beta_{\mathrm{value}}=$3.78$\pm.3$; p<.001 permutation test) and had no significant impact on preference learning (low cumulative: $\beta_{\mathrm{preference}}=1.60 \pm.12$; high cumulative: $\beta_{\mathrm{preference}}=1.87 \pm.1$4; p=.11 permutation test)

**Comment 1.4:** In the 'Testing Alternative Interpretations' section on Page 21, the manuscript tests whether there is a shift in value learning over the course of learning by means of a variant model in which there is a difference in the absolute value learning parameter only in the second half of training. This stepwise change model seems to me to be not giving a fair shot to this alternative explanation - what if the change is gradual, rather than occurring all of a sudden half way through the task? I suggest that a stronger test of this alternative explanation would be if the beta_preference and beta_value parameters were permitted to vary linearly across the course of training (with an intercept and slope fit to the data) rather than assumed to be constant or varying in a stepwise fashion.

**Response:** We agree that allowing beta_preference and beta_value to vary gradually across the duration of training would offer a more sensitive test of the duration of training. We originally implemented a stepwise effect because that best mirrors the way concurrent diversity was modelled, namely, as a categorical effect and not as an effect that develops gradually. This too, however, is only an approximation, since subjects do not know whether concurrent diversity is low or high from trial one, and even once they know, it unclear whether their mode of learning would change immediately. Thus, a fair comparison between the effects of training duration and of concurrent diversity requires allowing both effects to develop gradually.

To do this, we tested one model where the shift towards absolute-value learning occurs gradually over the course of learning, and compared it to a variation of our model where the effect of concurrent diversity on the balance between value and learning develops gradually.

Model comparison favored the latter model over both the training duration model (BIC +741) and our original model (BIC +1051). This indicates that there is a gradual shift between preference and value learning, but it proceeds in opposite directions under high and low concurrent diversity. We report these findings in the manuscript under ‘Testing alternative explanations’ (lines 317-328; new text in bold):

*A necessary consequence of varying concurrent diversity is an extension of the duration of learning, since twice as many trials are required to learn about twice as many images. This raises the possibility that it was simply the duration of learning, and not the diversity of learning exemplars, that shifted learning from relative preferences to absolute values.* ***To test this interpretation, we implemented a variant of our model where a shift towards absolute-value learning progresses gradually during learning irrespective of concurrent diversity. We compared this model to a matched implementation of a concurrent diversity effect, that is, where a gradually developing shift progresses towards value learning under high concurrent diversity but towards preference learning under low concurrent diversity. Model comparison favored the latter model over all other models (***$\boldsymbol{\Delta}$***BIC +741). This result indicates that shifts between preference and value learning indeed developed gradually. Most importantly, though, this result confirms again that the direction of the shift depended on concurrent diversity.***

We have added details of the implementation of the model in the Alternative Models subsection of the Methods (line 743-748):

*To test for a gradual shift towards absolute-value encoding as a function of time (see Ruling Out Alternative Explanations), we implemented a model that scales the inverse temperature parameter for value learning,* $\beta_{value},$ *by* $e^{\tau\cdot trial\left( i,t \right)}$*, and the inverse temperature parameter for preference learning,* $\beta_{Preference}$*, by* $\frac{1}{e^{\tau*trial\left( i,t \right)}}$*. Here τ is a free parameter controlling the degree of the shift, as a function of the number of trials that elapsed since image i was chosen at trial* $t$*. These inverse temperatures apply specifically to the outcome obtained that trial.*

## Reviewer 2

**Comment 2:** Solomyak and colleagues investigated the environmental conditions that promote the learning of absolute value or relative preferences among choice options, both of which were common strategies in humans’ decision-making. The article investigated four learning environments and tested the participants’ performance in both learned and novel pairs. Interestingly, the article found that training diversity promoted absolute-value-guided choice. However, a few major issues listed here needed to be addressed.

#### **Comment 2.1**: A major concern was whether the results of the current study could be explained by a third hypothesis: ranked preference. Specifically, the participants might learn the relative preference but also make use of the transitivity of relative preferences. That is, participants might learn the rank of items, but not necessarily the absolute value of each item. For example, if participants learned A was better than B and B was better than C in the training phases, then participants would easily infer A was better than C, even in situations where the participants did not learn the value of A and C directly. Whether there is evidence supporting the absolute value strategies that cannot be explained by this hypothesis?

**Response:** To examine whether there is evidence supporting the absolute value strategies that cannot be explained by transitive inference we isolated all novel-pair trials which had no transitive relation between them. On such trials, it would be impossible to make any indirect inferences about the relation between images.

By design, this includes all images learned in low concurrent diversity and a significant subset of images learned in high concurrent diversity. Across all conditions, subjects were significantly above chance 82% $\pm1$ in selecting the higher-value image, showing that transitivity of learning preferences cannot generally account for participants’ choices.

Furthermore, we have now calculated the generalization performance drop-off using only the non-transitive subset of trials. Consistent with our main findings, we find that concurrent diversity significantly improves generalization (p=.003, bootstrap test) within this subset of trials as well.

We now report these analyses in the main text (line 342-347):

*Finally, we examined an alternative hypothesis that participants make use of the transitivity of relative preference, thereby inferring a global rank of items without learning the expected value of each item. However, even among pairs of images with no transitive relation between them, subjects were significantly above chance in selecting the higher value image (*$Mean=.82 \pm.1$*). Moreover, the effects of concurrent diversity on generalization were significant within this subset of trials as well (p_corrected_ =.003 bootstrap test).*

**Comment 2.2:** A second concern was the contributing factor to the generalization effect. The article found the generalization effect by calculating the accuracy differences between the novel pairs and the learned pairs (Figure 3) It was not clear whether the generalization effect was caused by the higher performance in the novel pairs or lower performance in the learned pairs. It might be helpful to do further analysis to confirm the contributing factor to the generalization effect and report the effect size in all four conditions to exclude the possibilities that only some of the conditions contribute to the effect.

**Response:** We thank the reviewer for this suggestion. We have now evaluated whether the improved generalization under high concurrent diversity was primarily due to higher performance in novel pairs or lower performance in learned-pair trials. We found that neither significantly differed as a function of concurrent diversity, indicating that the difference in generalization emerged from their combination. This is to be expected because the ability to choose between learned pairs essentially constitutes an upper bound on the ability to choose between novel pairs. Indeed, these two types of performance were highly correlated (r = .77).We now note these results in the main text (line 149-151), where we reference the full breakdown of the results that is reported in Supplementary Table S1:

*This difference between low and high concurrent diversity was neither due to a difference in learned-pair trials nor in novel-pair trials (Table S1), but specifically reflected the drop-off in accuracy between them (p_corrected_ =.015 bootstrap test).*

**Table S1. Performance on testing trials.**

**Learned-pair test**

|  | **Mean** | **SE** | **Bootstrap-test** |
| --- | --- | --- | --- |
| **Low concurrent** | .87 | .02 |  |
| **High concurrent** | .86 | .02 | p=.42 |
|  |  |  |  |
| **Low cumulative** | .88 | .01 |  |
| **High cumulative** | .85 | .01 | p=.20 |

**Novel-pair test**

|  | **Mean** | **SE** | **Bootstrap-test** |
| --- | --- | --- | --- |
| **Low concurrent** | .82 | .01 |  |
| **High concurrent** | .84 | .01 | p=.10 |
|  |  |  |  |
| **Low cumulative** | .85 | .01 |  |
| **High cumulative** | .80 | .01 | p=.01 |

**Comment 2.3** It is not clear whether statistical tests in this article had been corrected for multiple comparisons (e.g., The test in Figure 3 needed to be corrected, since the same data was used to test the two hypotheses, concurrent diversity and cumulative diversity)

**Response:** We apologize for this omission. Multiple comparisons are performed in the manuscript whenever both concurrent and cumulative diversity are tested. We have now made sure that all such tests use a FDR-corrected threshold. This is now reported in the methods under ‘Statistical analysis’ (line 612-615):

*All non-modeling statistical analyses were performed in R using RStudio. Statistical tests were carried out using the bootstrap method with the “simpleboot” package. Correction for multiple comparisons across the two types of diversity was carried out using the Benjamini–Hochberg procedure.*

**Comment 2.4:** There are some typos in the manuscript: (i) In the caption of Figure 2, the t value was missing, “t(51.8)”; (ii) Figure citations need to be consistent (e.g., Line 113, “Figure 2b” to Figure 2B); (iii) Figure 4 was not cited in the main text.

**Response:** We thank the reviewer for pointing out these errors and have updated the manuscript accordingly.

**Comment 2.5:** Some preprint references has been published and might be updated. (e.g., “Hayden B, Niv Y: The case against economic values in the brain. PsyArXiv 2020”, now is Hayden BY, Niv Y. The case against economic values in the orbitofrontal cortex (or anywhere else in the brain). Behavioral Neuroscience. 2021;135: 192–201. doi:10.1037/bne0000448)

**Response:** We thank the reviewer for pointing out these changes and have updated the manuscript accordingly.

## Reviewer 3

**Comment 3.1**: The paper poses an excellent question and will be a good addition to the literature once the authors expand and clarify the methods. The analyses also need to be strengthened. At the moment, it is unfortunately difficult to ascertain the particulars the experimental procedure, which somewhat reduces the interpretability of the results. This is particularly worrying as we (this review is a joint work) work in the field of relative/absolute value learning.

**Response:** We thank the reviewers for the generally positive feedback and the many helpful suggestions that will help clarify the experimental procedure. Below we address each of the reviewer’s specific comments. Please see our detailed comments below:

#### **Comment 3.2:** Options/conditions. It is not very clear what options participants saw when and what was their visual representation. It would be helpful to have a figure similar to Figure 1C showing the option combinations for each day, condition, session and game, explicitly stating both the outcome probability of each option as well as the image (or image id) shown to the participant. Also note that Figure 1B&C suggests a different set-up of your task than I believe was the case. Namely, it suggests that in the low concurrent condition, games in the same session used the same options (and images) and that options/images were repeated across the conditions.

**Response:** We apologize for the confusion generated by our figure. To avoid further misunderstandings, we have now substantially modified the figure so as to include all the information requested (see new figure 1C). Among other changes, the figure now shows the actual images presented to subjects as well as the reward probability associated with each image across all four conditions.

**Figure 1. Experimental design. (A) Reward learning game.** On each trial, subjects were asked to choose one of two circular images. Following their choice, subjects either received or did not receive a reward of 1 coin based on a fixed reward probability associated with the chosen image (0, 1/3, 2/3, or 1). Each game consisted of 48 such ‘learning’ trials, interleaved with 24 ‘testing’ trials wherein subjects chose between images about which they learned in prior sessions. Outcomes were not revealed in testing trials to prevent further learning. Every image first appeared in 64 learning trials over two sessions before subjects were tested on it. ITI: inter-trial-interval. **(B) Daily schedule.** Each day, subjects performed two experimental sessions on a specially designed mobile phone app (Eldar et al., 2018), one in the morning (on average, at 8:56 am, and no earlier than 6:00 am) and one in the evening (on average, at 6:12 pm, and no earlier than 4:00 pm). In each session, subjects played two games in which they learned about a total of six images. **(C)** **Experimental conditions.** Four experimental conditions were implemented along 10 days of learning, each lasting two to three days. Each condition is illustrated via a representative selection of six pairs of images subjects chose between in two different sessions. Within the low concurrent diversity condition (left two columns), three images were learned over the span of each game, whereas in the high concurrent diversity (right two columns) six images were learned over the span of two games. **Thus, in both conditions, each image appeared in 32 learning trials per session**. In the low-cumulative-diversity conditions, each image was pitted against the same two images **in two consecutive learning sessions**, whereas in the high-cumulative-diversity conditions, images were pitted against different images **(sometimes in two nonconsecutive sessions; see methods for details).** Three days of training, as opposed to two, were required for high-cumulative-diversity conditions so that each image could be pitted against different images in its two learning sessions. Conditions were randomly ordered, and equal in terms of average reward probability and number of images learned per session. Testing trials involved choosing between two images the subject already chose between during learning (**Learned-pair trials, 25% of testing trials)** or novel pairings of images learned separately **(Novel-pair trials, 75 % of testing trials). On average, pairs were tested 27** $\boldsymbol{\pm.8}$ **times.**

#### **Comment 3.3:** Experimental testing. Very little is said about the particulars of the testing trials. Can you please include more details? Here are some of the questions we had:

**Response:** We thank the reviewers for pointing out details that were missing or unclear. Below we provide answers to each of their questions, and we note where in the manuscript each of the missing details can now be found. Here, we reproduce the Experimental Design section text that has been amended accordingly (lines 535-553, changes in bold):

*To assess the formation of absolute values, we had subjects choose between images about which they had learned in two previous sessions (‘testing’ trials). Through the entire course of learning, such testing trials were interleaved with the learning trials (every 3rd trial,* *24 testing trials total). Reward feedback was not shown on testing trials, but subjects were informed in advance that these trials would be rewarded with the same probabilities with which they were rewarded during learning. This reward was factored into the final bonus subjects received for their performance. Testing trials always presented a choice between images learned within the same condition. However, some of these images subjects had already chosen between during learning (‘Learned Pair Trials’; 25% of testing trials), whereas other trials presented a choice between images about which subjects learned in separate games (‘Novel Pair trials’; 75% of testing trials). Half of novel-pair trials were designed to assess how well subjects performed in general. These trials thus presented a choice between two images one which of was preferable to the other both in terms of reward probability and in terms of how it ranked in reward probability compared to the other images it was learned with. The other half of novel-pair trials were designed to distinguish between value and preference learning. Thus, half of these (25 % of all novel-pair trials) presented a choice between images with the same expected value but that ranked differently in their original learning context, whereas the other half presented a choice between images with the same relative rank but different values. Pairs that satisfied these criteria were selected in random.*

**Comment 3.3.1:** What was the ratio of learned vs novel pairs?

**Response:** About a quarter of testing trials (25%) were learned-pair trials, whereas the rest (75%) were novel-pair trials. We included more novel-pair trials since we were interested in separately estimating performance on different sub-types of these trials (see response to **Comment 3.3.4** below). This is now clarified in the Experimental Design subsection of the Methods (lines 543-544)

**Comment 3.3.2:** How many repeats were there per pair?

**Response**: On average, there were 27 $\pm.8$ repeats per pair during testing. This is now reported in the legend to Figure 1C.

**Comment 3.3.3:** Was day 11, the only day you tested images from multiple conditions or was this also the case on previous days? Were the paired options originally from the same day/condition or did you combine options across day/conditions?

**Response**: Prior to day 11, within every learning session, we tested only the latest condition for which at least two learning sessions were completed. This meant that in most days only one condition was tested. However, equalizing the total number of testing trials across conditions required that there be on average 2 days (range 1-3) in which the morning and evening sessions tested different learning conditions. The variation between subjects emerged because of Sabbath observance, which resulted in some subjects completing only the morning session on Fridays (since sundown prevented the completion of the second session) and either subsequently continuing the following evening (Saturday evening) or the following day (Sunday morning). We now report these details in the methods section (lines 555-562).

**Comment 3.3.4:** How did you come up with the novel pairs? Did you have any criteria when drawing the novel pairs or were the pairings random, or exhaustive (all possible combinations)?

**Response****:** Half of novel-pair trials were designed to assess how well subjects performed in general. These trials thus presented a choice between two images one which of was preferable to the other both in terms of reward probability and in terms of how it ranked in reward probability compared to the other images it was learned with. The other half of novel-pair trials were designed to distinguish between value and preference learning. Thus, half of these (25 % of all novel-pair trials) presented a choice between images with the same expected value but that ranked differently in their original learning context, whereas the other half presented a choice between images with the same relative rank but different values. Pairs that satisfied these criteria were selected in random. These details are now better clarified in the methods section (lines 544-553).

**Comment 3.3.5:** While feedback was not shown for the testing trials, were the testing trials rewarded? And if so how?

**Response:** Subjects were informed in advance that testing trials would be rewarded with the same probabilities with which they were rewarded during learning. This reward was factored into the final bonus subjects received for their performance. This is now clarified in the methods section (line 536-540).

**Comment 3.3.6** Also there is some information that you mention in the figure legend but not in the methods themselves. Could you please put all relevant info in the methods even if it means repeating it? E.g. Figure 1C - There were 24 testing trials per game

**Response:** All information in the legend is now included in the Methods in the Experimental Design subsection.

##### **Comment 3.4:** Rank bias by group. It would be great if you could split novel pairs testing into groups based on the relative and absolute value prediction and show the bias separately for each group. In particular the following grouping will be of particular interest:

**Comment 3.4.1:** A group with options that have the same absolute value but differ in the relative value (I believe you already isolated those and plotted them in Figure 4A?)

**Response:** Indeed, we tested and plotted the results for this group in Figure 4A. To recap, we show that, under low concurrent diversity subjects preferred images with a higher relative value. We report these results in detail in the Results section lines 185-191.

###### **Comment 3.4.2:** A group with pairs where the relative value is the same (or very similar) for both options but their absolute value is different, e.g. options like 100% vs 66% from low reward context and 66% from high reward context vs 33% from low reward context

**Response:** Per reviewer suggestion, we isolated testing trials where both options ranked similarly compared to the images with which each was learned, but their absolute value is different. We found that subjects were significantly more accurate on such trials in which the images had been learned under high, as compared to low, concurrent diversity (bootstrap test p=.035). By contrast, subjects were significantly less accurate for images learned under high, as compared to low, cumulative diversity (bootstrap test p=.02), as consistent with the generally reduced performance found under high cumulative diversity. This result further strengthens the conclusion that concurrent diversity promotes value learning. We now report it in the Results section (lines 170-183; new text in bold):

*By contrast, relative preferences also account for the outcomes of other images against which the presently available images were pitted during learning. These latter outcomes determined how each of the available images ranked compared to other images during learning. Due to accounting for these outcomes, a relative preferences learner should* *show no preference between images with similar rankings during learning even if their absolute values differ,* *but favor similarly rewarded images that ranked higher in their original learning context (i.e. a rank-bias).*

*In examining choices between similarly ranked images with different absolute values, we found that concurrent diversity improved subjects’ accuracy (Figure S1;* $Mean\_high=.83\pm.02$*;* $Mean\_low=.78\pm.02$*; p_corrected_ =.035 bootstrap test), as consistent with a shift towards value learning. By contrast, cumulative diversity impaired performance on such trials (*$Mean\_low=.82\pm.02 Mean\_high=.77\pm.02$ *p_corrected_ =.02 bootstrap test), as consistent with its general detrimental effect on overall performance.*

**Figure S1: Choice accuracy on similarly ranked images with different expected values across diversity conditions.** Concurrent diversity improved subjects’ performance ($Mean\_high=.83\pm.02$; $Mean\_low=.78\pm.02$; p_corrected_ =.035 bootstrap test). By contrast, cumulative diversity impaired performance on such trials ($Mean\_low=.82\pm.02 Mean\_high=.77\pm.02$ p_corrected_ =.02 bootstrap test), as consistent with its general effect on overall performance. The plot shows individual subject accuracy (circles), group distributions of accuracy levels (violin), group means (thick lines) and standard errors (gray shading).

###### **Comment 3.4.3:** A group where one of the options has both higher relative and absolute value, e.g. 100% vs 33% low reward context. If I understood your set-up correctly, this should be the majority of novel pairs.

**Response:** Indeed, in most novel-pair trials one of the images had both higher relative and absolute value than the other. In these trials, there was no significant effect of concurrent diversity (p=.22), and there was a trend for subjects to perform worse on images learned in high, as compared to low, cumulative diversity (p=.052 bootstrap test). Together with the previous results, this shows that concurrent diversity predominantly affected trials that specifically probe absolute value learning. This analysis is now reported in the Results section (line 223-231).

###### **Comment 3.4.4:** Potentially, also a group where the option with the higher absolute value has lower relative value (though I don’t think there was any pair like this in your task.

**Response:** The reviewer is correct; we did not have such pairs in our task.

##### **Comment 3.5:** Did you observe any difference in responses to the testing trials across the span of the experiment? Did participants become more absolute encoders over time as they learned that they will be tested on novel pairs (as they did in Juechems 2021)

**Response:** Unlike in Juechems et. al. 2021, our subjects were made aware from the beginning of the experiment (during their initial lab visit) that they would need to generalize learned values. Therefore, we did not expect to see a gradual shift towards value learning as reported in that paper.

Nevertheless, we have now tested for a gradual shift by fitting the data with a variant of the computational model where the balance between value and preference learning was allowed to gradually change over the course of the experiment. This model did not fit the data well as the original model which did not assume a gradual shift (+BIC 353). Thus, as expected, we did not find evidence that subjects gradually adjusted their strategy irrespective of task condition over the course of the experiment. We now report this result in Alternative Models (lines 750-759).

*To additionally determine whether our data might be better accounted for by prior work which suggested that humans gradually shift towards value learning in similar RL experiments (Juechems et al., 2022), we implemented an alternative model which assumes that the shift towards absolute-value encoding grows with the number of trials that elapsed since the beginning of the experiment. This model, too, did not fit the data well as the original model that did not assume a continuous gradual shift (+BIC 353), likely because subjects were made aware from the beginning of the experiment that they would need to generalize learned values.*

##### **Comment 3.6** In choice sets where the 100% option was present, how often participants explored the alternative option.

**Response:** On average, subjects explored the alternative option 11% during learning. By the end of learning, measured as the last five feedback trials, subjects selected against the 100% option 5% of the time. This low level of exploration is consistent with subjects knowing that reward probabilities did not change. During testing, subjects selected against the 100% option on 8% of trials. Note that these latter choices do not constitute exploration because outcomes were not revealed.

**Comment 3.7:** What about the simulations of the alternative models? Are they falsified by the data?

**Response:** To further substantiate our claims that our winning model is best able to fit the data we simulated data from the main alternative models, each implemented with parameter settings that were fitted to subjects’ choices, and we then compared the simulated and actual data.

**Preference-only model**

We simulated data from a variant of our model that only learns relative preferences. To recap, this model does generalize relative preferences since it also learns how well each image faired generally relative to the other images with which it was learned. Nevertheless, this model was unable to account for subjects’ ability to perform generalization at a high level across conditions, thus severely underestimating subjects’ performance on those trials. Posterior model prediction: mean=.66 $\pm.1$ vs Real data mean=.83$\pm.2$.

**Value-only model**

We also simulated data from a variant of our model that only learns expected values. Such a model is well suited to generalize to novel-pairs but fails to account for the difference in accuracy between learned-pair and novel-pair trials ($Mea{n\Delta}_{concurrent low}=0 \pm.1,Mea{n\Delta}_{concurrent high}=0 \pm.1$;$p=.55 bootstrap$). Furthermore, such a model is unable to account for the effects of other images’ outcomes on image choices. Thus, in the data simulated using this model the effect of other images’ outcomes was not significant CI=[-5.31,4.84] nor was it modulated by concurrent diversity CI=[-4.45,5.76].

In sum, neither value learning nor preference learning alone could account for subjects’ behavior. We now include this information in the results section (294-299) and elaborate on these model falsifications in the Falsification of alternative models subsection of the Methods (lines 806-816):

*We found that the full model accounted for subjects’ choices across both learning and testing trials significantly better than the alternative models (Figure 5; Table S3). Moreover, only the full model was able to recreate in simulation all the behavioral findings, including generalization performance, effect on choice of outcomes for other images, and rank bias (see Figure S2 and Falsification of alternative models in Methods).*

*Falsification of alternative models*

*In addition to model comparison, we examined whether alternative models generate predictions that are falsifiable by the data. Simulations from a Preference-only model could not account for subjects’ ability to perform generalization at a high level across conditions (Posterior model prediction: mean=.66* $\pm.1$ *vs Real data mean=.83*$\pm.2$*.), whereas a value-only model could not account for the difference in accuracy between learned-pair and novel-pair trials*

*(*$Mea{n\Delta}_{concurrent low}=0 \pm.1,Mea{n\Delta}_{concurrent high}=0 \pm.1$*;*$p=.55 bootstrap$*). Furthermore, such a model was unable to account for the effects of other images’ outcomes on image choices. Specifically, the effect of other image’s outcomes was not significant CI=[-5.31,4.84] nor was it modulated by concurrent diversity CI=[-4.45,5.76]. Thus, neither value learning nor preference learning could account for subjects’ behavior alone.*

**Comment 3.8:** (lines 182 - 184) to prove your point, you should test that preference for higher ranked images in the low concurrent condition was significantly higher than in the high concurrent condition (and the same for the cumulative condition). If one group is significantly different from a reference value while the other group is not, it does not mean that the group difference is significantly different

**Response:** We agree with the reviewer’s assessment and now also report the results of a direct comparison between the raw numbers and report as follows (line 190 *This difference between conditions trended towards significance (p=.06 permutation test).* We note though that this analysis is primarily meant for visualization since it is limited in sensitivity and validity. For this reason, conclusions are drawn based on the subsequent regression analysis. See full details in the response to Comment 1.2 above.

**Comment 3.9:** (lines 147 - 154). How did you test this?

**Response:** We specify our calculation of the rank bias beginning with line 627 in the Methods and have updated the legend of Figure 4A in the main text to clarify our definition of rank:

**Figure 4A Legend**

***Rank bias as a function of training diversity.*** *Y-axis shows the percent of trials involving similarly rewarded images in which subjects chose the image that ranked higher during learning.* ***A subject’s ranking of each image was based on how many times they chose the image relative to the other images it was pitted against (best, second-best, or worst, see Methods for more details).*** *The plot depicts individual subjects’ choices (circles), group distributions (violin), group means (thick lines) and standard errors (gray shading).*

**Methods**

*To calculate whether subjects preferred images ranked higher in the original learning context,* *we calculated each subject’s ranking of each image based on how many times they chose the image relative to the other images it was pitted against (best, second-best, or worst). If the difference in choice frequency between two images that were pitted against each other was below 10%, indicating no established ranking between them, then the pair was excluded from the analysis (8% of trials). These rankings were then averaged across the two learning sessions in which an image was learned about to generate an overall rank for the image. Finally, we tested for a ranking bias by examining subjects’ choice between differently ranked but similarly rewarded images (defined as less than a 10% difference in percent rewarded outcomes). Bias was defined as a tendency to choose the image that had been ranked higher during learning.*

**Comment 3.10:** The authors should refer to Haynes and Wedell (“in and out of context”; Journal of Experimental psychology).

**Response:** We thank the reviewers for bringing to our attention this important paper. We now reference it in our introduction of preference learning (line 49-50):

**Comment 3.11:** The authors should mentioned within lines 51-52 “authors have proposed models of” a reference to Palminteri and Lebreton 2021, where most of the relative value learning models are summarized.

**Response:** We thank the reviewers for bringing to our attention this important review and have now cited it as suggested.

**Comment 3.12:** Even more importantly, the proposed winning model is a variant of the model proposed by Bavard et al. (2018; Nature Communications; HYBRID), however somehow surprisingly this is not acknowledged and the paper is not cited.

**Response:** We agree the model that best fitted our data is essentially a **variant** of the Bavard et al 2018 model, and we now acknowledge this in the main text (line 243-245):

*To do this, we fitted subjects' choices during both learning and testing with a computational model that combines value and preference learning (as proposed by Bavard et al., 2018).*

**Comment 3.13:** Bavard et al. (2021; Science Advances) propose a model to explain why context effects (i.e., deviation from absolute values) are stronger in blocked concerning interleaved design (the idea is global versus local context values). Is this idea relevant here?

**Response:** Our results are consistent with Bavard et al 2021 findings that blocked design promotes learning of relative values. However, we extend these prior findings by showing that relative preferences emerge even when the range of outcomes is fixed across contexts (lines 382-391).

*Our findings agree with prior work showing that emphasizing comparisons between a limited number of specific images, for instance by repeatedly presenting subjects with a choice between the same two options and providing reward information about the foregone option, promotes learning of relative values (Bavard et al 2021). However, the process by which the formation of relative values in the latter experiments has so far been explained – namely, normalization to the range of outcomes experienced during learning – cannot explain relative preference learning in our experiment. This is because the range of outcomes in our experiments was the same in all learning sessions.*

**Comment 3.14:** The fraction of your code that is publicly available is written in MATLAB as opposed to R you mentioned in the paper. - - Did you do part of modeling in MATLAB? If so, can you specify what optimizer (and other functions) you used?

**Response:** We apologize for this omission. All relevant code is now publicly available on our github account. We specify that we fit model parameters to subjects' choices using an iterative hierarchical importance sampling approach (Eldar et al 2018) using MATLAB scripts available at <https://github.com/eeldar/model-fitting> (line 763)

**Comment 3.15** The distinction you make between concurrent and cumulative conditions was not very clear from the introduction. Although, Figure 1 somewhat clarified the issue, rephrasing the relevant lines in the introduction (72-75) would be beneficial, alternatively adding an example might also help.

**Response:** We thank the reviewers for this suggestion and have updated the manuscript accordingly with the following text (line 74-78, changes in bold).

*These considerations suggest at least two types of training diversity may support and incentivize value learning. The first type of diversity relates to how many options a person learns about* ***concurrently within a given learning session*** *(henceforth, concurrent diversity), whereas the* ***second type of diversity is the number of alternative options a given option is cumulatively pitted against, across all learning sessions*** *(henceforth, cumulative diversity).*

We have also modified figure 1C (see response 3.2 pg. 9) to better clarify the distinction between concurrent and cumulative diversity.

**Comment 3.16:** Figure 1A - there is no ITI in the picture, I believe that a game had 48 trials instead of the 72 mentioned in the figure.

**Response:** In figure 1A the very first column displays ITIs. Every game had 48 feedback trials and 24 no-feedback trials totaling 72 trials. We clarify this information in the legend of Figure 1A as well as in the methods (line 476,537)

**Comment 3.17:** 109-111 – This is calculated on data from the learning trials right? I.e. trials with feedback

**Response:** The reviewer is correct, this result reflects overall accuracies from learning trials. We have now edited the main results to clarify this (line 109).

**Comment 3.18:** 129 – not sure I understand what a proportion reward difference is in this case. Can you please elaborate?

**Response:** We apologize for the lack of clarity. For each image, we calculated how frequently it was rewarded when chosen during learning (i.e., number of times rewarded divided by number of times chosen). The absolute difference between the reward rates of the two images available on a given testing trial offers a measure of trial difficulty. We have now clarified this in the manuscript (line 122-126, changes in bold):

*Novel and learned pairs involved the same images and presented subjects with similarly difficult choices, in the sense that the time elapsed since learning was roughly the same (novel pairs = 1.88 days* $\pm$*.08, learned pair=1.75* $\pm$*.08 following learning),* ***as was the average difference in reward rate between the two images that made up a pair*** *(reward rate difference:* $\Delta_{novel-pair}$*=48.4%* $\pm2\%$*,* $\Delta_{learned-pair}=49.1\% \pm3\%)$*.*

**Comment 3.19:** - 179 – p=.006 is missing a period before the 00

**Response:** We have corrected this error.

**Comment 3.20:** - 184 – If you stated in the previous section that cumulative conditions had no effect on absolute vs relative learning. Why is it suddenly problematic that you do not find any effect of the cumulative condition on a related measure.

**Response:** We agree this is not problematic. The point we were trying to make is that it is all the more convincing that cumulative diversity did not enhance value learning, because it did not reduce the performance drop-off even though there was another, unrelated reason it could have had. Namely, the fact that *pairs of images from conditions of high cumulative diversity were encountered during learning half as many times as pairs of images in low-cumulative-diversity conditions* (line 154-157).

**Comment 3.21:-** 268 – Is the βvalueV(i) + βpreference’(i) ever over 1?

**Response:** Β_valueV(i) and β_preferenceW’(i) were treated as distinct inverse temperature parameters, and thus were both fitted to values higher than 1, as consistent with the level of choice stochasticity that best fitted the data. We now clarify this in describing our model (lines 310-312, changes in bold):

*Importantly,* $\beta_{value}$ *and* $\beta_{preference}$ *are distinct inverse temperature parameters,* ***the respective magnitudes of which determine the degree to which absolute values and relative preferences influence choice.***

**Comment 3.22:** 282-284 - β preference is stated twice with different results. I assume one of them was supposed to be β value?

**Response:** We apologize for this error and have now corrected it in the manuscript.

**Comment 3.23: -** 279 - Did you fit the model only on the learning data or also on the testing data?

**Response:** We fit all models on both the learning and the testing data and have edited the Results section to clarify this (line 301).

**Comment 3.24: -** 327- Did you get the same results with AIC?

**Response:** To address the reviewers’ question, we examined the AIC of the relevant models, and now report the findings in Supplementary Table S5 (reproduced below), which we reference in the Model Comparison subsection of the Methods (line 787).

**Table S5: Model comparison using AIC.** To validate the BIC model comparison results, we also performed model comparison using the Akaike information criterion (AIC).

|  | **AIC** | **BIC** |
| --- | --- | --- |
| **Winning model** | 56,576 | 56,854 |
| **Value-only model** | 59,352 | 59,482 |
| **Preference-only model** | 66,476 | 66,624 |
| **RW variant of the winning model** | 57,478 | 57,755 |

**Comment 3.25: -** Supplementary Table 2 - Low Concurrent - Day 2 - Game 1 - Wasn't this supposed to be {4,5,6} and {10,11,12}? as per the previous schedule?

**Response:** The purpose of that table (reproduced below for reference) was to demonstrate how stimuli were arranged in conditions of high cumulative diversity. The arrangement ensured that every image would be pitted against four distinct images across two learning sessions and thus we could not pair {4,5,6} together in Day 2 because 4 and 5 were already paired in Game 1 of Day 1’s evening session. Similarly, 10 and 11 were previously paired in the evening session of Game 2 (day 1) and therefore cannot be paired again. By pairing {2,5,6} in Game 1, we ensured that stimulus 2 (previously paired with 1,3), 5 (previously paired with 1 and 4), and 6 (only later paired with 3 and 4) all were pitted in four distinct pairings. We have now modified the legend of the table to clarify this rationale (the legend and the ‘Low concurrent’ section of this table are reproduced below).

**Table 2: Example arrangement of images across days in conditions of high cumulative diversity.** Each number corresponds to an image subjects learned about. **The arrangement ensured that every image would be pitted against four distinct images across two learning sessions**. Brackets group images that were pitted against each other. **A) Low concurrent.** Each game consisted of three images which were all pitted against each other. **B) High concurrent.** Each game consisted of six images, but every image was only pitted against two other images across trials.

**Low concurrent**

| **Day 1** | **Day 2** | **Day 3** |
| --- | --- | --- |
| **Morning session** | | |
| **Game 1:** {1,2,3} | **Game 1:** {2,5,6} | **Game 1:** {14,15,18} |
| **Game 2:** {7,8,9} | **Game 2:** {8,11,12} | **Game 2:** {13,16,17} |
| **Evening session** | | |
| **Game 1:** {1,4,5} | **Game 1:** {3,4,6} | **Game 1:** {13,14,17} |
| **Game 2:** {7,10,11} | **Game 2:** {9,10,12} | **Game 2:** {15,16,18} |

**Comment 3.26:** - 539 - Did you observe differences in performance in the test trials during the regular 10-day schedule and during day 11 (i.e. after one day break?) How many people took the break for holiday observance, did it fall within or between conditions and did it affect the performance in any way?

**Response:** Subjects showed no significant difference in performance on day 11 testing trials (.84 $\pm.1$) relative to the other testing days (.86$\pm.1$ p=.14 bootstrap test)

25/27 subjects took a break and 56% of breaks occurred within condition. Breaks were distributed across conditions in the following way:

Concurrent Low, Cumulative Low: 20 %

Concurrent Low, Cumulative high: 36 %

Concurrent High, Cumulative Low: 20 %

Concurrent High, Cumulative High: 24 %

Out of the 25 subjects that took a break, 6 of them took the break in the midst of learning about specific images (they had only completed one of two learning sessions with those images). We evaluated whether these breaks resulted in accuracy drop-off for these stimuli relative to other stimuli within the same condition for which learning was uninterrupted. We found no significant drop-off ($Mean_{with break}=.87\pm.04, Mean_{without break}=.84 \pm.03$;$p=.196$,bootstrap test$).$

We now report these results in the Daily Schedule subsection of the Methods (lines 595-602).

*Twenty five out of twenty-seven subjects took a holiday break, but only six of those subjects took the break during learning about specific images (they had only completed one of two learning sessions with those images). We evaluated whether these breaks resulted in accuracy drop-off for these images relative to other images within the same condition for which learning was uninterrupted but found no significant drop-off (*$Mean_{with break}=.87\pm.04, Mean_{without break}=.84 \pm.03;p=.196$*bootstrap test).*

**Comment 3.27:** 878 - Can you include the model you used to calculate the rank bias?

**Response:** We were unsure as to what the reviewer was referring to here since there was no line 878 in the manuscript. However, we clarify how rank bias was calculated and tested in response to comment 3.7 above.

**Comment 3.28:** 635 - choice difference**?**

**Response:** The choice difference refers to the difference between stimuli in how many times they were previously chosen during feedback. We have clarified the manuscript accordingly.

**Comment 3.29:** 644 - Could you make the gap between the first fraction and the second fraction larger? On a first read it looks like a single fraction.

**Response:** We have adjusted the fraction displayed accordingly.

**Comment 3.30:** 653 - Can you include a full description of the RW model you used, including free parameters?

**Response:** In order to test whether a beta-binomial model was best suited to explain subject choices, we compared it to a Rescorla Wagner model which also learned expected values and relative preferences with all of the main effects and interactions included in our winning model. The only parameters that differed between models were the RW learning rates $\alpha_{value},\alpha_{preference}$, which replaced the leak parameters $\gamma_{value},\gamma_{preference}$ included in the winning model. This is now detailed in our Alternative Models section (736-741).

**Comment 3.31:** Did you also use R for the model fitting? IF you which package?

**Response:** As clarified above in response to Comment 3.14, we fit model parameters to subjects' choices using an iterative hierarchical importance sampling approach (Eldar et al 2018) using MATLAB scripts available at <https://github.com/eeldar/model-fitting>

## Reviewer 4

**Comment 4**: Solomyak et al present an interesting and ambitious experiment testing how two different sources of diversity, concurrent and cumulative, push participants to learn the expected or relative value of a choice over 10 days. They found that concurrent, but not cumulative, diversity helps participants encode the expected versus relative value of an option by enabling better generalization when making new choices between previously learned options. Nevertheless, this manuscript could substantially benefit from major revisions. Namely, it is challenging to adequately assess the results and their interpretation when the task details are unclear.

**Response:** We thank the reviewer for the helpful suggestions for how to revise the manuscript and make the task details and results clearer. Below we address each of the reviewer’s specific comments.

##### **Comment 4.1:** I appreciate that this is a complicated task to describe, but it is currently very difficult to understand task details and conditions, and in some cases, task visualization appears to conflict with task description.

— For example, (line 482) in the low concurrent diversity condition, two games within each learning session are said to each have a distinct set of three images, yet in the experimental design figure (figure 1), it appears that they share the same three images. While I understand that the figure may not describe exact task conditions, it is extremely confusing and should mirror the actual task as much as possible. From the figure, one would infer that some options (i.e., orange and green) are sampled every day, across conditions and much more frequently than other options (i.e., hot pink and light green).

**Response:** We apologize to the reviewers for the confusion that Figure 1 generated. We have re-designed the figure to better clarify the details of the experimental design. As the revised figure hopefully makes clear, we used unique images for each condition. The figure is reproduced above in response to comment 3.2 (p. 9).

**Comment 4.3:** From the methods, it appears that there were 60 stimuli, did participants learn all 60?

**Response:** All subjects learned all 60 stimuli. This information is now reported in line 480.

**Comment 4.4:** How many times did they get to see each stimulus?

**Response:** Each stimulus was viewed 64 times with feedback. This information is now reported in line 480.

**Comment 4.5:** Over how many days was each stimulus presented? I know the latter differs on whether cumulative diversity was high or low, but even in the table example on page 32, it appears that some stimuli are repeated across days, and some are not – how was this determined. These task details need to be made much clearer and in the main manuscript (the experimental design figure should also include this information).

**Response:** We apologize for the lack of clarity and thank the reviewer for helping us clarify the experimental protocol. Each stimulus was learned in two sessions. All stimuli learned in low cumulative diversity conditions were learned in two consecutive learning sessions over the course of a single day. In high cumulative diversity conditions, pairing each image with different images in two sessions meant that, for some stimuli, its two sessions could not be consecutive, and thus these stimuli were learned over more than one day. To be precise, 58.33% of high cumulative diversity were learned over the course of two days.

This extension of the learning period conferred a small benefit to accuracy in testing trials, as shown by a logistic regression on the number of days learning spanned (log odds accuracy improvement=.12 CI=[.05 .19]). Reassuringly, this incidental effect ran counter to the overall effect of high cumulative diversity, which was to impair testing performance. Thus, it does not change the interpretation of the main results.

We have now added the relevant information to Figure 1’s legend in the main manuscript (reproduced above in response to comment 3.2) and we report the effect of multiple-day learning in the Methods section (beginning line 525):

*Implementing these criteria made it impossible to have subjects learn about twelve images in the span of two days, and thus, we had subjects learn about 18 images across the span of 3 days (Table 2). Furthermore, pairing each image with different images in two sessions meant that, for some stimuli, its two sessions could not be consecutive. Thus, 58.33% of high cumulative diversity were learned over the course of two days.*

*This extension of the learning period conferred a small benefit to accuracy in testing trials, as shown by a logistic regression on the number of days learning spanned (log odds accuracy improvement=.12 CI=[.05 .19]). Reassuringly, this incidental effect ran counter to the overall effect of high cumulative diversity, which was to impair testing performance. Thus, it did not change the interpretation of the main results.*

##### **Comment 4.6:** (line 508) It is mentioned that for high cumulative diversity, the reward context was the same in both learning sessions of a given image. Was this true of other conditions? What was the high/low reward context breakdown by condition/day? (could also be noted in experimental design).

**Response:** Indeed, the reward context was the same in both learning sessions of a given image across all four conditions (stated in lines 524-526). Within each day, an equal number of images were learned in the high and low reward contexts (lines 497-498).

##### **Comment 4.7:** How were the novel pairs chosen?

**Response:** Half of novel-pair trials were designed to assess how well subjects performed in general. These trials thus presented a choice between two images one which of was preferable to the other both in terms of reward probability and in terms of how it ranked in reward probability compared to the other images it was learned with. The other half of novel-pair trials were designed to distinguish between value and preference learning. Thus, half of these (25 % of all novel-pair trials) presented a choice between images with the same expected value but that ranked differently in their original learning context, whereas the other half presented a choice between images with the same relative rank but different values. Pairs that satisfied these criteria were selected in random. These details are now better clarified in the methods section (beginning with line 543).

###### **Comment 4.8**: It is mentioned that the stimuli were paired with stimuli from prior sessions (‘two sessions’ back), but was this restricted to the same condition?

**Response:** Yes, testing trials always paired images learned within the same condition. We now specify this information in Experimental Design (line 555).

###### **Comment 4.9**:Additionally, how were the novel pairs chosen on the last day? I see they were drawn from different conditions, did this lead to different choice behavior than during interleaved testing? How did older versus newer options fare on Day 11 testing? (i.e., how did long-term memory influence choice?).

**Response:** We apologize for the confusion regarding day 11 testing. The morning session of day 11 exclusively tested stimuli from the last learned condition. This was needed to ensure that stimuli learned on days 9 and 10 would be tested as many times as stimuli learned on previous days. In the evening session, subjects were presented with testing trials that spanned across conditions. However, not all subjects performed these trials, and some performed them incompletely. Therefore, the data from the evening trials were not included in the main analyses.

Nevertheless, we have now examined whether performance on the morning session of day 11 significantly differed from performance on the other days. Subjects showed no significant difference in performance on day 11 testing trials (.86 $\pm.1$) relative to the other testing days (.84$\pm.1$ p=.14 bootstrap test) suggesting that interleaving testing and learning trials did not adversely affect performance. Additionally, we examined whether older images fared worse on day 11 testing using a Bayesian logistic mixed model and found no significant effect of time since learning on testing trial performance (log odds increase in likelihood of accurately choosing=-0.03 CI=[-0.10,.04]).

##### **Comment 4.10:** To rule out the potential confound that a difference in the duration of learning is modulating the high versus low concurrent condition effects, authors tested a model where the shift towards absolute value learning only occurred in the second half of high-concurrent sessions. Since this model fit worse, they ruled out the confound. While I agree with this approach and interpretation, it belies an interesting assumption that more learning should lead to more ‘absolute’ versus relative preference. From what I can glean from the manuscript, the full model doesn’t seem to incorporate a dynamic “shift” from relative to absolute values, right? I say this because of the large literature on habits showing that, conversely, it could be expected that more learning leads to stronger habits/relative preference. Perhaps the potential/speculated dynamic between expected and relative value learning could be expanded upon in the discussion. Alternatively, if there is a way to test a shift from relative to absolute value learning in the current data, that should be examined.

**Response:** We thank the reviewer for this insightful suggestion. To test this idea, we developed a variation of our model where the effect of concurrent diversity on the balance between value and preference learning develops gradually. We compared this model both to the original model and to a model where a gradual shift towards value learning developed gradually irrespective of condition.

Model comparison favored a gradual effect of concurrent diversity on the balance between preference and value learning (BIC +741). This indicates that there was a gradual shift between preference and value learning, but it proceeded in opposite directions under high and low concurrent diversity. Thus, more learning led to more habit-like preference learning only under low concurrent diversity. Conversely, high concurrent diversity gradually shifted learning towards absolute value learning. We report these findings in the main text under ‘Testing alternative explanations’ (lines 317-328). Additional details of these analyses and excerpts of new text can be found above in the response to Comment 1.4.

##### **Comment 4.11:** A fascinating tension in the concurrent condition is that while (as expected) learning is worse in the high condition, generalization is better. Is this observed at an individual-difference level? Are worse learners better able to generalize? It could be an interesting consequence of this trade-off

**Response:** We thank the reviewer for this interesting idea. To determine whether worse learners were better at generalizing, we evaluated whether there was an inverse correlation across subjects between learning performance and the generalization performance drop-off. This correlation, however, was not significant (r=. -0.12 [-.48,.27]). We surmise that the inverse relationship between learning and generalization may be specific to the effects of concurrent diversity.

**Comment 4.12:** To differentiate independent versus relative value and given the literature on learning ‘extreme’ values within a learning context, were there test trials between the ‘extreme’ options in the two reward contexts? i.e., comparing choice between (1) stimulus associated with 0 reward (low-reward context) and stimulus associated with 0.33 probability reward in the high-reward context and (2) stimulus associated with 0.66 reward probability in low-reward context versus the 100% probability reward stimulus (high-reward context). If so, is there a reliable difference in choice for these pairs in the different conditions?

**Response:** To answer this question, we examined choices between the poorest, or between the best, options of the low and high contexts. Evaluating subject accuracy on these trials we found reliable differences in accuracy between the low (.83$\pm.03$) and high (.9$\pm.02$) concurrent diversity conditions. This result further supports the hypothesis concurrent diversity promotes absolute value learning. In the revised manuscript, this result now forms part of a slightly more inclusive analysis which generally targets choices between images with the same rank but different value. See the response to Comment 3.4.2 above for further details and new supplementary Figure S1.

**Comment 4.13:** Equations 6 and 7 are difficult to read. For equation 6, you may want to have consistent notation with the paragraph above (a/b versus i/j), and I can’t parse equation 7, there seem to be missing/inconsistent notation of operations.

**Response:** We apologize to the reviewer for the difficult to parse notation. We have adjusted equation 6 to have consistent notation as follows

$P\left( \mathrm{choice}=i \right) \sim$ ${\sigma(\beta_{0} + \beta}_{\mathrm{Own}}Own_{A-B}+ \beta_{Current alternative}{Current Alternative}_{A-B} {+ \beta}_{\mathrm{Other}} Other_{A-B} +$

$\beta_{Times Chosen}Times Chosen_{A-B}+ \beta_{\mathrm{Concurrent}}Concurrent +$

$\beta_{\mathrm{Cumulative}}Cumulative + \beta_{Concurrent \times Own}Concurrent\times Own_{A-B}+$ $\beta_{Concurrent \times Current alternative}Concurrent\times{Current Alternative}_{A-B}+$

$\beta_{Concurrent \times Other}Concurrent\times Other_{A-B} + \beta_{Cumulative \times Own} Cumulative\times Own_{A-B}+$ $\beta_{Cumulative \times Current alternative}Cumulative\times{Current Alternative}_{A-B}+$

$\beta_{Cumulative \times Other}Cumulative\times Other_{A-B} )$	*(equation 6)*

Furthermore, equation 7 has been re-written as follows:

$\beta_{preference=}\beta_{preference-baseline}\times\beta_{\frac{\mathrm{low}}{\mathrm{high}}\mathrm{concurrent}}\times\beta_{\frac{\mathrm{learning}}{\mathrm{testing}}}\div\beta_{\frac{\mathrm{low}}{\mathrm{high}}\mathrm{cumulative}}\times\beta_{\frac{\mathrm{low}}{\mathrm{high}} cumulative \times\frac{\mathrm{value}}{\mathrm{preference}}}\div\beta_{\frac{\mathrm{value}}{\mathrm{preference}}\times\frac{\mathrm{learning}}{\mathrm{testing}}}\div\beta_{\frac{\mathrm{low}}{\mathrm{high}} cumulative\times\frac{\mathrm{learning}}{\mathrm{testing}}}\div\beta_{\frac{\mathrm{low}}{\mathrm{high}} concurrent \times\frac{\mathrm{value}}{\mathrm{preference}}}\times\beta_{\frac{\mathrm{low}}{\mathrm{high}} concurrent\times\frac{\mathrm{learning}}{\mathrm{testing}}}$

$\beta_{value=}$ $\beta_{value-baseline} \times\beta_{\frac{\mathrm{low}}{\mathrm{high}}\mathrm{concurrent}}\div\beta_{\frac{\mathrm{low}}{\mathrm{high}}\mathrm{cumulative}}\times\beta_{\frac{\mathrm{learning}}{\mathrm{testing}}}\div\beta_{\frac{\mathrm{learning}}{\mathrm{testing}}\times\frac{\mathrm{low}}{\mathrm{high}}\mathrm{cumulative}} \div\beta_{\frac{\mathrm{low}}{\mathrm{high}}\mathrm{cumulative} \times\frac{\mathrm{value}}{\mathrm{preference}}} \times\beta_{\frac{\mathrm{learning}}{\mathrm{testing}}}\beta_{\frac{\mathrm{low}}{\mathrm{high}}\mathrm{concurrent}}\times\beta_{\frac{\mathrm{low}}{\mathrm{high}} concurrent \times\frac{\mathrm{value}}{\mathrm{preference}}}\times\beta_{\frac{\mathrm{value}}{\mathrm{preference}}\times\frac{\mathrm{learning}}{\mathrm{testing}}}$	*(equation 7)*

**Comment 4.14:** It appears that only some of the effects of the Bayesian logistic mixed model (equation 6) are reported, it would be helpful to have a full table or report of the results.

**Response:** We have added the full table below and have included it in the manuscript (Table S2)

**Table S2. Bayesian logistic regression of subjects’ choices on reward history, training diversity, and past choice.**

|  | Estimate | Est. Error | l-95% CI | u-95% CI |
| --- | --- | --- | --- | --- |
| $\beta_{0}$ | -0.02 | 0.04 | -0.10 | 0.06 |
| $\beta_{Own}$ | 1.97 | .16 | 1.63 | 2.31 |
| $\beta_{Current alternative}$ | -.43 | .11 | -.64 | -.21 |
| $\beta_{\mathrm{Other}}$ | -.53 | .10 | -.75 | -.32 |
| $\beta_{\mathrm{Concurrent}}$ | .01 | .02 | -.04 | .05 |
| $\beta_{Times Chosen}$ | .04 | .01 | .04 | .05 |
| $\beta_{Concurrent \times Own}$ | .17 | .09 | -.10 | .44 |
| $\beta_{Concurrent \times Current alternative}$ | .06 | .06 | -.08 | .20 |
| $\beta_{Concurrent \times Other}$ | .39 | .05 | .30 | .49 |
| $\beta_{\mathrm{Cumulative}}$ | -.01 | .02 | -.04 | .02 |
| $\beta_{Cumulative \times Own}$ | -.21 | .07 | -.34 | -.06 |
| $\beta_{Cumulative \times Current alternative}$ | -.06 | .06 | -0.17 | .05 |
| $\beta_{Cumulative \times Other}$ | .11 | .07 | -.02 | .23 |

**Comment 4.15:** The description of “own” versus “current alternative” and “other” isn’t clear or very intuitive. I think it would help to have a clear example of the three calculations and their distinctions.

**Response:** Following the reviewer’s suggestion, in the revised manuscript, we now provide concrete examples of the three calculations and their distinctions (beginning with line 638, new text in bold):

*The predictors are described below for a choice between image A and image B:*

1) $Own$ *- Reward history of the currently available images in the current pair, computed as the proportion of rewarded trials when option A was chosen versus any image minus the proportion of rewarded trials when option B was chosen versus any image*$.$
2) $Current Alternative$ *- Reward history of the currently available images in the previous times the same images were pitted against each other, computed as the proportion of rewarded trials when option A was rejected in favor of option B minus the proportion of rewarded trials when option B was rejected in favor of option A.*
3) $Other$ *- Reward history of choosing against the currently available images, computed as the proportion of rewarded trials when option A was rejected versus any image other than B minus the proportion of rewarded trials when option B was rejected versus any image other than A.*

*To provide a concrete example, consider the following five-trial segment for which we calculate the corresponding value of each regressor:*

*Trial 1: A vs B, A is selected and rewarded*

*Trial 2: B vs C, B is selected and is not rewarded*

*Trial 3: B vs C, C is selected and rewarded*

*Trial 4: A vs C, A is selected and is not rewarded*

*Current trial. Trial 5: A vs C*

*“Own” is calculated as the proportion of times A was rewarded when A was chosen (Trial 1, Trial 4 for a total of A_own=1/2) minus the proportion of times image C was rewarded when chosen (Trial 3; C_own=1/1). Thus,* $\beta_{own}=\frac{1}{2}-1=-\frac{1}{2}.$

*“Current alternative” is calculated as the proportion of times the subject was rewarded when they rejected image A in favor of image C (no such trial exists so A_current alternative is set to the null value of 1/2), minus the proportion of times the subject was rewarded when they rejected image C in favor of image A (trial 4 – C= 0). Thus* $\beta_{current alternative}=1$

*“Other” is calculated as the proportion of times the subject was rewarded when they rejected image A in favor of any image other than C (no such trials exist so A_other=1/2) minus the proportion of times the subject was rewarded when they rejected image C in favor of any image other than A. In our case, image C was rejected in favor of image B (trial 2) and image B is not rewarded (0/1) so the value of* $\beta_{other}=\frac{1}{2}$

**Comment 4.16:** The language describing “absolute” values could be slightly misleading since “absolute” is often used to mean unsigned (i.e., a value of 5 and -5 both have an absolute value of 5). I don’t think you need to change this language throughout the paper but comparing “expected (or ‘independent’) value” and “relative value” may be more straightforward.

**Response:** We agree with the reviewer and thus we now clarify the meaning of “absolute” value in the introduction (line 20, changes in bold).

*A large body of decision-making research suggests that humans learn from experience* ***the expecte****d value of different available options* ***(hitherto referred to as these options’ absolute values)***

**Comment 4.17:** It is first said that consecutive sessions were separated by 12 hours on average, but later (line 547) it appears this difference was (at least 6) and closer to 9 hours, and the average hours are also different in the experimental design figure description (6:56 versus 6:12).

**Response:** We thank the reviewer for pointing out these inconsistencies in the manuscript, which we have now corrected (the correct time was 6:12 pm). The 12-hour figure included the time difference between the evening session and the morning session the following day. To avoid confusion, we have now revised the Daily Schedule subjection of Methods to read (line 592-593):

*On average, subjects performed the morning session at 8:56AM (mean SD ± 40 min) and the evening session at 6:12 PM (mean SD± 32 min).*

**Comment 4.18:** I think there is a typo in Table 1 C) for stimulus 5 - it says it’s pitted against 3 and 7, but stimulus 7 isn’t included in that set.

**Response:** We thank the reviewer for catching this typo. The reviewer is correct, and the table has been fixed as follows:

**C) Concurrent diversity:** High; **Reward context:** Low

| **Stimulus** | **Expected value** | **Pitted against** | **Optimal choice frequency** |
| --- | --- | --- | --- |
| **1** | 0 | 2 and 3 | Never |
| **2** | .33 | 1 and 4 | Half (over 1) |
| **3** | .66 | 1 and 5 | Always |
| **4** | .66 | 2 and 6 | Always |
| **5** | .33 | 3 and **6** | Half (over 6) |
| **6** | 0 | 4 and 5 | Never |

**Comment 4.19:** (line 65) “Unfamiliar sets of familiar options” may be better understood as “New or novel sets” of learned or experienced options.

**Response:** We agree with the reviewer’s suggestion and have updated the manuscript accordingly.
